# Outcomes of the gastrocnemius flap performed by orthopaedic surgeons in salvage revision knee arthroplasty

**DOI:** 10.5194/jbji-6-425-2021

**Published:** 2021-11-23

**Authors:** Robert Allan McCulloch, Amirul Adlan, Scott Evans, Michael Parry, Jonathan Stevenson, Lee Jeys

**Affiliations:** 1 Royal Orthopaedic Hospital, Bristol Road South, Northfield, Birmingham, B31 2AP, UK; 2 Aston Medical School, Aston University, Birmingham, UK; 3 School of Life and Health Sciences, Aston University, Birmingham, UK

## Abstract

**Introduction**: The gastrocnemius myofascial flap is used to manage soft-tissue defects over the anterior aspect of the knee in the context of a patient presenting with a sinus and periprosthetic joint infection (PJI) or extensor mechanism failure. The aim of this study was twofold: firstly, to evaluate the outcomes of gastrocnemius flaps performed by appropriately trained orthopaedic surgeons in the context of PJI and, secondly, to evaluate the infection-free survival of this patient group. **Patients and methods**: We retrospectively reviewed 30 patients who underwent gastrocnemius flap reconstruction during staged revision total knee arthroplasty for prosthetic joint infection (PJI). All flaps were performed by an orthopaedic surgeon with orthoplastics training. Patients had a mean age of 68.9 years (range 50–84) and were followed up for a mean of 50.4 months (range 2–128 months). A total of 29 patients (97 %) were categorized into Musculoskeletal Infection Society (MSIS) local extremity grade 3 (greater than two compromising factors), and 52 % of PJIs were polymicrobial. The primary outcome measure was flap failure, and the secondary outcome measure was recurrent infection. **Results**: Flap survival was 100 % with no failures or early returns to theatre for flap problems such as necrosis or haematoma. Overall infection-free survival during the study period was 48 % (13 of 27 infected cases). Using limb salvage as the outcome, 77 % (23 of 30 patients) retained the limb. Infection recurrence occurred in 48 % (10 patients) in the type B3 cohort and 67 % (4 patients) in the type C3 cohort ( p=0.65 ). **Conclusions**: The surgical technique for a gastrocnemius myofascial flap is reliable and reproducible when performed by appropriately trained orthopaedic surgeons, even in high-risk groups. However, the risks of recurrent infection and amputation remain high within our series due to poor host and extremity factors.

## 1 Introduction

Prosthetic joint infection (PJI) is a serious complication after knee arthroplasty which represents a significant clinical and economic burden for surgeons, healthcare systems and, most importantly, patients (Haddad, 2017; Kurtz et al., 2007; Lenguerrand et al., 2017a). PJI results in poor function and is associated with an increased mortality at 5 years (Lum et al., 2018). The decreasing age of patients undergoing primary arthroplasty and the increasing rates of revision mean a greater need for revision arthroplasty due to PJI. The number of patients undergoing multiple revision procedures is also increasing, and within this patient group, there is an increased risk of PJI compared to primary arthroplasty (Anon, 2021). The risk of PJI after aseptic revision knee replacement is approximately 3 % at 10 years compared to 0.8 % at 10 years in primary TKR (total knee replacement; Huotari et al., 2015; Lenguerrand et al., 2017b). PJI patients with concomitant soft-tissue defects and extensor mechanism deficiencies are known to have higher rates of reinfection and amputation (Bickels et al., 2001). Soft-tissue reconstruction is essential to manage the infection and achieve limb salvage (Xu et al., 2019). Various methods of soft-tissue coverage around the knee have been described; however the gastrocnemius flap provides anterior knee coverage with minimal donor site morbidity and without microvascular surgery (Harrison et al., 2018; Theil et al., 2020). Due to the dissection required, the flap can be relatively easily learned with a rapid learning curve (Shahzad et al., 2016). The gastrocnemius rotational flap is indicated for defects that cannot be closed primarily after appropriate debridement of a sinus and therefore resulting in exposed metalwork and subsequent infection. The flap is best suited for defects distal to the patella, due to the confines of the rotational length of the flap which is based on its proximal pedicle.

Within the United Kingdom, a gastrocnemius flap is typically performed by plastic surgeons. However, elsewhere this is a technique performed by orthopaedic surgeons with specific training in orthopaedic oncology or microvascular techniques (Malawer and Price, 1984; Tetreault et al., 1999). Due to the increasing complexity of salvage surgery in PJI, this procedure is becoming an essential part of revision knee arthroplasty (Malawer and Price, 1984; Tetreault et al., 1999). All senior authors performing gastrocnemius flaps within this study have completed fellowship training in orthopaedic oncology and use this technique during oncological reconstructions.

The published literature does not distinguish a difference in outcomes regarding flap failures and complications when comparing procedures performed by orthopaedic surgeons or plastic surgeons (Malawer and Price, 1984; Osinga et al., 2020; Tetreault et al., 1999). This has the advantage of minimizing the resource burden as a single surgical team is required for both the orthopaedic and plastic surgical steps of the procedure. The limitation of orthopaedic surgeons solely managing soft-tissue defects is that they may not have capabilities to perform other procedures if a gastrocnemius flap is inadequate to provide coverage or if it has subsequently failed. This reiterates the importance of a multidisciplinary approach to the management of this complex patient group whether or not joint orthopaedic and plastic surgical teams are required for individual cases. The aim of this study, therefore, was to answer the following questions: firstly, whether, in the context of salvage revision knee surgery, appropriately trained orthopaedic surgeons can safely perform gastrocnemius flaps and, secondly, what the limb salvage rates are for patients presenting with PJI requiring a gastrocnemius flap.

## 2 Patients and methods

The study involved a retrospective analysis of all revision knee surgeries presenting to a high-volume revision arthroplasty centre. Patients were identified from a prospectively collated infection database. After local approval, a retrospective collection of all patients who had undergone a gastrocnemius flap coverage whilst undertaking a revision knee arthroplasty was performed. All oncological indications were excluded. Between 2012 and 2020, a total of 30 patients underwent a gastrocnemius flap during revision knee surgery performed by four orthopaedic surgeons. Patients undergoing endoprosthetic reconstructions and arthrodesis reconstructions for salvage of infected knee arthroplasties were excluded. Demographic data were retrospectively collected, along with operative details and technical specifications of the flap (medial gastrocnemius, lateral gastrocnemius or bilateral). Patients' past medical history and microbiology results were evaluated. The presence of multidrug-resistant (MDR) organisms was recorded. Patients were categorized according to their general health and limb status based on the Musculoskeletal Infection Society (MSIS) scoring system (Parvizi et al., 2018).

Follow-up was defined as the time from flap reconstruction to last clinical review. Primary endpoint measurement was flap failure. Flap failure was defined as a requirement to return to theatre for a reason specifically related to the gastrocnemius flap or flap necrosis managed non-operatively as per previous publications on this topic (Xu et al., 2019). Secondary outcomes were recurrence of infection and amputation. Failure was defined as recurrent infection or relapse involving the same or a different microorganism using the MSIS criteria (Atkins et al., 1998) and those who underwent resection arthroplasty or amputation or whose death was related to the infection. The length of follow-up was taken from the date of first operation to treat the PJI to the date of last clinical review.

The indication for the gastrocnemius flap in three patients was extensor mechanism reconstruction; therefore they were excluded from the infection-free survival analysis as all were aseptic on sampling.

**Table 1:** Patient demographics and surgical information.

Variables	N	%
Female	18	60
Male	12	40
ASA (American Society of Anesthesiologists) score		
2	18	60
3	12	40
MSIS category (host, extremity)		
B3	23	77
C2	1	3
C3	6	20
Flap		
Medial	24	80
Lateral	4	13.3
Both	2	6.7
Indication		
Sinus	27	90
Extensor mechanism reconstruction	3	10
Timing of reconstruction		
First-stage revision	21	70
Debridement and implant retention	4	13.3
Single stage	4	13.3
Second stage	1	3.3
Definitive implants		
TKR	1	3
Revision TKR	10	30
Endoprosthetic replacement	9	30
Arthrodesis prosthesis	4	13
Did not progress to second stage (static spacer)	6	20
Previous PJI treatment	16	59 (16 of 27)
Multiple revisions	19	63 (19 of 30)

The remaining 27 patients had a gastrocnemius flap due to a sinus. The mean patient age was 68.9 (range 50–84). Mean follow-up was 50.4 months (range 2–128). According to the MSIS staging system, 97 % (29 patients) were categorized into local extremity grade 3 (greater than two compromising factors). Patient demographics including full MSIS staging and surgical data are displayed in Table 1. A total of 20 of 30 (67 %) patients had previously undergone at least one revision surgery, and 63 % (17 of 27 patients) had previously failed a revision procedure for PJI. A proportion of 80 % (24 patients) had an isolated medial gastrocnemius flap, 13 % ( n=4 ) had an isolated lateral gastrocnemius flap and 6 % ( n=2 ) had both lateral and medial gastrocnemius flaps. A total of 21 cases (70 %) of the gastrocnemius flaps took place during the first stage, four cases (13.3 %) as part of a single stage revision for infection, four cases (13.3 %) during debridement and implant retention procedure (DAIR) and one case (3.3 %) during second-stage revision for extensor mechanism reconstruction.

## 3 Treatment

All patients were managed under the BIS (Bone Infection Service) multidisciplinary team, where both the surgical and microbiological management is discussed. Two-stage revision for PJI remains the standard of practice within our department for cases with a sinus requiring flap reconstruction, and a static spacer is constructed for stability of the soft tissues.

Four patients underwent single rather than staged flap reconstruction, two were aseptic extensor mechanism reconstructions and two underwent single stage revision with flap reconstruction due to medical co-morbidity and frailty. The other extensor mechanism reconstruction took place after an acute traumatic extensor mechanism failure with subsequent DAIR procedure which proved to be negative on intraoperative sampling.

**Figure 1:**
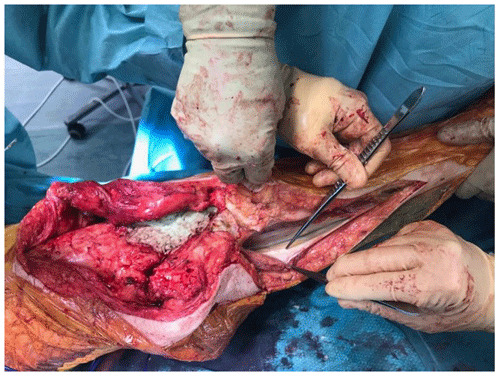
Extension of incision to identify the medial gastrocnemius muscle belly.

**Figure 2:**
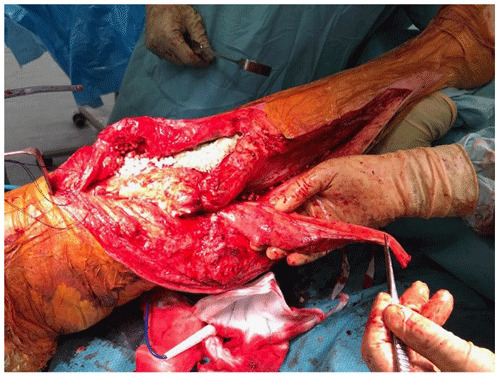
Medial gastrocnemius flap raised.

**Figure 3:**
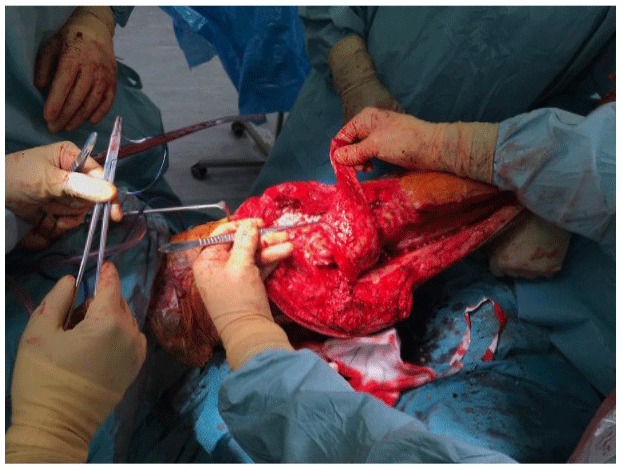
Inlay of the flap to cover the soft-tissue defect.

At the time of the procedure, a minimum of five samples are sent as per the established sampling technique (Atkins et al., 1998). Samples are sent for microbiological culture for a minimum of 7 d. After initial debridement and explantation, the midline incision is extended distally along the medial border of the tibia (Fig. 1). The posterior compartment fascia is incised. The gastrocnemius muscle is identified, with anatomical landmarks being the sural nerve and the plantaris tendon. The sural nerve acts as the midline structure and a guide to the raphe. On the superficial aspect of the muscle, meticulous haemostasis needs to be performed due to perforating vessels that can cause a post-operative haematoma. The medial gastrocnemius muscle is raised at the musculotendinous junction with the tendo-Achilles and elevated proximally (Fig. 2). The amount of proximal extension of the flap will be dependent on the excursion required to cover the soft-tissue defect. Inlay of the flap is performed once the cement spacer has been inserted (Fig. 3). Raising the flap prior to insertion of a static spacer is preferable as having some bend in the knee can aid dissection. Once the flap is secured, a split-thickness skin graft from the ipsilateral thigh is applied. If the gastrocnemius flap is being used for an extensor mechanism reconstruction, a portion of the tendo-Achilles is harvested with the flap and then weaved into the patellar tendon remnant. Following fabrication of a static antibiotic loaded cement spacer (ABLCS), the flaps are secured to the joint capsule and the split skin graft applied on top. Post-operative protocol is incisional negative pressure dressings for 7 d and assessment at this stage then conversion to traditional dry dressings. An extension splint is used for at least 2 weeks. Mobility is resumed after 7 d with protected weight-bearing status. All patients are reviewed in clinic at 6 weeks post-operatively. All patients are reviewed by our outreach nurses post-operatively until day 31 in the community with feedback on any wound or clinical concerns to the senior clinicians.

Raising the flap at second-stage re-implantation is usually straightforward after identifying the lateral border of the flap (if it is a medial gastrocnemius flap) and using the plane between the flap and the tibial periosteum to elevate medially.

## 4 Statistical analysis

All statistical analyses were performed with SPSS Statistics 24.0 (IBM, Armonk, New York, USA). Median and mean values with ranges were calculated for continuous variables. Kaplan–Meier survival curves were generated with R (Vienna, Austria) to assess overall survival, and a log-rank test was used to assess statistical significance. A chi-squared test was used to test statistical significance for categorical variables. A p value of <0.05 was set to be statistically significant.

**Figure 4:**
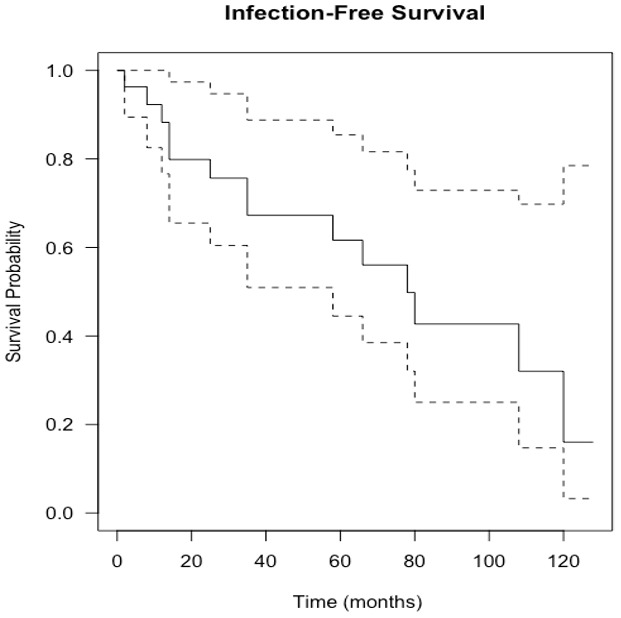
Kaplan–Meier curve showing infection-free survival following knee revision surgery with gastrocnemius flap for infection eradication. The dashed lines show 95 % confidence intervals.

**Figure 5:**
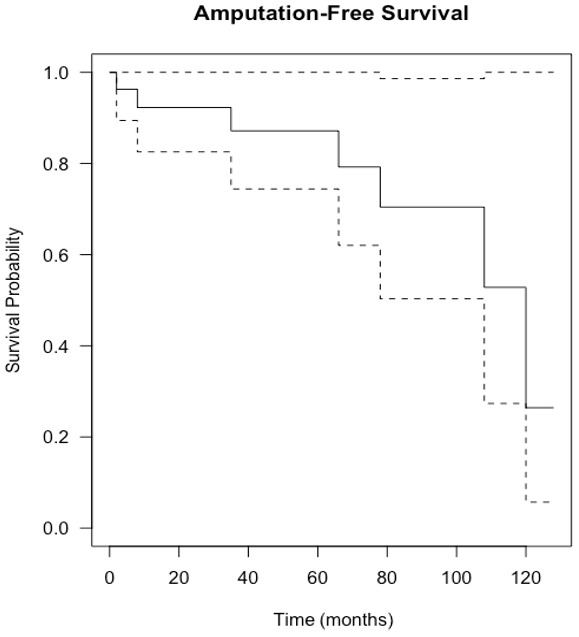
Kaplan–Meier curve showing amputation-free survival following knee revision surgery with gastrocnemius flap for infection eradication. The dashed lines show 95 % confidence intervals.

**Figure 6:**
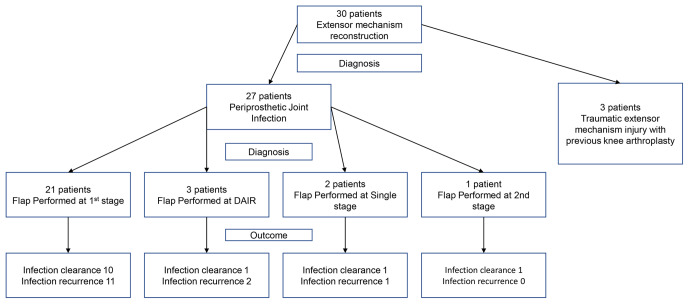
Flow diagram to represent patient procedures and outcomes for infection clearance.

## 5 Results

Flap survival was 100 % with no failures or early returns to theatre for flap problems such as necrosis or haematoma or skin graft failure at all time points. Overall infection-free survival during the study period was 48 % (13 of 27 infected cases). A summary of patient procedures and outcomes can be seen in Fig. 6. A summary of the microbiological results for the patients can be seen in Table 3. The estimation of infection-free implant survival was 80 % (confidence interval (CI) 65 %–97 %) after 2 years and 62 % (CI 45 %–85 %) after 5 years (see Fig. 4). Using limb salvage as the outcome, a total of 77 % of patients retained their limb during the study. The amputation-free survival after 2 years was 92 % (CI 83 %–100 %) and 87 % (CI 74 %–100 %) after 5 years (see Table 2 and Fig. 5).

**Table 2:** Patient outcomes during study period.

Outcome	n	%
Salvage	23 (no amputation)	76
Further surgery procedures excluding amputation	8	27 (8 of 30)
Long-term antibiotic suppression	9	33 (9 of 27)
Amputation	7	23

**Table 3:** Microbiological results (of 27 infected cases).

Organism	Number of patients
Polymicrobial	15
*S. aureus*	15
*S. epidermidis*	5
*Candida* sp.	3
Culture negative	2
*Lactobacillus*	1
*Rothia kristinae*	1

A total of 21 patients (70 %) underwent flap reconstruction during first-stage surgery, with the remainder undergoing flap reconstruction as part of a DAIR procedure. Within this group, two were due to a post-operative fall from their index surgery, resulting in an open-wound dehiscence and resultant soft-tissue defect. In the other two patients, they had a history of multiple revisions and endoprosthetic reconstruction that presented with acute infection and sinus formation.

When comparing patients with and without a prior history of PJI, the infection-free survival was 81 % (CI 60 %–100 %) after 12 months and 61 % (CI 37 %–100 %) after 5 years. The infection-free survival rate for the group with a previous history of PJI was 79 % (CI 60 %–100 %) at 12 months and 63 % (CI 41 %–96 %) after 5 years. There was no statistically significant difference between the two groups ( p=0.82 ). Patients in the B3 category showed an infection-free survival rate of 90 % (CI = 78 %–100 %) at 12 months and 67 % (CI = 48 %–93 %) at 5 years. Patients in the C3 category with greater than two compromising systemic and local extremity factors showed infection-free survival of 83 % (58 %–100 %) at 12 months and 42 % (CI 15 %–100 %) after 5 years. Nevertheless, there was no statistically significant difference between the two categories ( p=0.099 ).

Six patients did not proceed to second-stage re-implantation and were managed definitively with a static ABLCS reinforced with Küntschner nails. One patient did not proceed to second-stage implantation due to anaesthetic concerns. In two patients, this was patient choice due to adequate function, rather than due to septic or aseptic failure, and did not require suppressive antibiotic therapy. Three patients suffered septic failure after first-stage revision and ABLCS, of which one patient underwent amputation due to uncontrolled infection and two were commenced on lifelong suppression and did not proceed to reconstruction nor repeat first-stage revision. A total of seven patients (23 %) proceeded to an amputation due to relapse of infection during the study period. Of the patients managed with amputation, six patients had a background of multiple revisions, and six patients had a multidrug-resistant infection. Five of the patients had a trial of suppressive therapy which had subsequently failed.

## 6 Discussion

Patients with PJI of the knee and resultant soft-tissue defects can be appropriately managed by trained orthopaedic surgeons performing pedicled gastrocnemius flaps and skin grafts.

Our cohort did not suffer flap failure; however this study highlights the high rates of recurrence of infection in this group of patients due to the poor host and extremity criteria of the patient group. In this study, 97 % (29 of 30) patients were categorized into local extremity grade 3 according to the MSIS staging criteria with greater than two compromising local tissue factors which increase the risk of re-infection (McPherson et al., 2002). The presence of a sinus is often associated with poor host factors and triples the rate of failure following two-stage revision compared to patients without a sinus (Xu et al., 2019). Within this paper, patients presenting with a sinus were more likely to be smokers or hypoalbuminaemic and to have had previous revision surgery. Therefore, the presence of a sinus both indicated poor host factors and higher failure rates with revision surgery. Our series' overall infection-free survival after 5 years was 62 % (CI 45 %–85 %).

Despite a high level of PJI recurrence, no patient within the series required further soft-tissue management, requiring the input of a plastic surgeon. This outcome, in the opinion of the authors, highlights one benefit of concentration of such complex patients in specialist prosthetic infection centres.

This large series included a majority of patients with previous revisions and multiple co-morbidities; 56 % of patients had a polymicrobial (PMR) infection, a greater proportion than previous literature, even in patients presenting with a sinus (Marculescu and Cantey, 2008; Tan et al., 2016). A sinus is an independent risk factor for a polymicrobial infection, and with that there is an increased risk of a failure of treatment; indeed, Parvizi et al. (2018) reported an odds ratio of 3.80 for amputation in PMR PJIs compared to monomicrobial infection (Tan et al., 2016).

A total of 52 % of patients had multidrug-resistant (MDR) organisms, which is high compared to the published literature (Siljander et al., 2018). Multidrug-resistant organisms are most frequently acquired secondary to multiple courses of antibiotics and procedures. Most of the patients in the present study had previous revisions and prolonged antibiotics for PJI eradication; thus a high proportion of patients had multidrug resistance. Worldwide there has been an increase of PJI from resistant organisms, and this has a deleterious effect on their outcomes (Tan et al., 2016).

High rates of amputation and infection persistence in this group are supported by previous publications in this field and raise larger questions regarding the management of the multiply revised PJI. Patients presenting with recurrent PJI should be made aware of the high rates of treatment failure and amputation risk.

Despite this, the authors would support limb salvage wherever possible as the functional outcomes for above-knee amputation in an elderly cohort remain poor (Sierra et al., 2003). The proportion of patients walking with a prosthesis after above-knee amputation following a knee PJI is estimated to be only 50 % (Fedorka et al., 2010). The psychological impact of an amputation and chronic PJI can also not be underestimated (Kunutsor et al., 2017). Success of limb salvage after revision TKR with flap coverage for PJI in the literature has been quoted to be 63 % at 5 years (Xu et al., 2019).

The mortality in this patient group during the study period was 17 %. This echoes recent evidence from Lum et al. (2018) of a 5-year mortality of 21.64 % with PJI of the knee (Kurtz et al., 2018). Our patient group contains a number of patients with multiple co-morbidities and provides evidence assisting in the appropriate education and consenting of patients undergoing PJI management.

The limitations of this study are its retrospective nature from a single centre and lack of functional scores for the patients. Although our sample size is small, it is comparable to previously published literature on the topic. We do not have a comparator group of patients where the gastrocnemius flap has been performed by plastic surgeons as within our institution this has historically been a procedure performed by orthopaedic surgeons.

Four patients had a follow-up period of less than 24 months. Of these patients, two had persistent infection and proceeded to an amputation. Due to this paper reporting on the outcomes of gastrocnemius flaps as its primary outcome, the authors felt that including these cases was relevant despite the relatively short follow-up period. There were no flap failures in our series and no returns to theatre post-operatively.

Although the series is from a single department, there are four surgeons within the department who performed the procedure, further reinforcing this as a safely reproducible technique. With the centralization of PJI management, the authors would suggest that the gastrocnemius flap may well become part of the armamentarium of revision knee surgeons with time. The authors' preference for a single-incision approach for both the knee revision procedure and the gastrocnemius flap provides added simplicity compared to a two-incision procedure and added utility in case of requirement for extensile approaches for supplementary procedures such as tibial tubercle osteotomy. A gastrocnemius flap raised with a skin paddle (medial sural artery perforator gastrocnemius flap) has been well described in the literature for coverage of soft-tissue defects around the knee secondary to trauma and infection (Ling et al., 2018). This removes the concerns of applying a split-thickness skin graft over the knee, potentially providing superior functional and aesthetic outcomes. However, within the technique described within this paper, due to a single-incision approach, this is not feasible. The authors would recommend that surgeons wishing to become trained in this technique should undertake additional training and that the institution in which the surgeon is working must also be skilled in the management of flap care post-operatively.

## 7 Conclusions

The gastrocnemius myofascial flap using a single incision provides reliable and reproducible soft-tissue coverage when performed by appropriately trained orthopaedic surgeons without flap failure. Patients with infected knee arthroplasties and soft-tissue defects suffer a high risk of recurrence of infection and subsequent amputation due to poor host and local tissue factors.

## Data Availability

Underlying research data can be accessed by contacting the corresponding author at robert.mcculloch@nhs.net.
